# VANLO - Interactive visual exploration of aligned biological networks

**DOI:** 10.1186/1471-2105-10-327

**Published:** 2009-10-12

**Authors:** Steffen Brasch, Lars Linsen, Georg Fuellen

**Affiliations:** 1 Department of Mathematics and Computer Science, Ernst-Moritz-Arndt-University, Greifswald, Germany; 2 School of Engineering and Science, Jacobs University, Bremen, Germany; 3 Institute for Biostatistics and Informatics in Medicine and Ageing Research, University Rostock, Germany

## Abstract

**Background:**

Protein-protein interaction (PPI) is fundamental to many biological processes. In the course of evolution, biological networks such as protein-protein interaction networks have developed. Biological networks of different species can be aligned by finding instances (e.g. proteins) with the same common ancestor in the evolutionary process, so-called orthologs. For a better understanding of the evolution of biological networks, such aligned networks have to be explored. Visualization can play a key role in making the various relationships transparent.

**Results:**

We present a novel visualization system for aligned biological networks in 3D space that naturally embeds existing 2D layouts. In addition to displaying the intra-network connectivities, we also provide insight into how the individual networks relate to each other by placing aligned entities on top of each other in separate layers. We optimize the layout of the entire alignment graph in a global fashion that takes into account inter- as well as intra-network relationships. The layout algorithm includes a step of merging aligned networks into one graph, laying out the graph with respect to application-specific requirements, splitting the merged graph again into individual networks, and displaying the network alignment in layers. In addition to representing the data in a static way, we also provide different interaction techniques to explore the data with respect to application-specific tasks.

**Conclusion:**

Our system provides an intuitive global understanding of aligned PPI networks and it allows the investigation of key biological questions. We evaluate our system by applying it to real-world examples documenting how our system can be used to investigate the data with respect to these key questions. Our tool VANLO (Visualization of Aligned Networks with Layout Optimization) can be accessed at .

## 1 Background

### 1.1 Introduction

In many biological processes proteins play a key role. They are involved in biological regulation, development, growth, locomotion, metabolism, and reproduction. Therefore, the study and analysis of proteins is of high importance in the fields of biology and medicine. Due to their chemical structure proteins are able to interact with each other. These interactions trigger many biological processes. For example, signals from the exterior of a cell are mediated to the interior of the cell by *protein-protein interaction *(*PPI*) of the signaling proteins. Such processes are also involved in diseases such as cancer. PPIs are fundamental to life, and their investigation yields insight into the evolution of animals [1] and into biochemical function [2].

For each species its proteins and their interactions form a PPI network. The PPI networks of different species are related if they evolved from a common ancestor whose PPI network can be viewed as their common ancestral network. Learning more about the evolution of PPI networks helps us understand the networks themselves. PPI networks can be aligned by finding proteins with the same common ancestor, so-called *orthologs *[3,4]. Investigation of such an alignment allows for the detection of similarities and dissimilarities between different species. For example, the interaction network between key regulators of stem cell pluripotency (the proteins Oct4, Sox2, and Nanog) is believed to be shared by mouse and human, while there are differences in the signaling network that controls the key regulators [5]. In Section 1.2 we provide the fundamental biological background on proteins, PPI networks, and their alignment. This description leads to the formulation of the key questions that one wants to address by investigating aligned biological networks.

Since tackling these questions requires the simultaneous exploration of different types of relationships between proteins, research on biological networks demands the support of a graphical display of such networks. As biologists are interested in viewing the interaction of the proteins within one species, but also the alignment based on the orthologous proteins between the species, standard graph layouts are of limited use. First attempts to the visualization of aligned biological networks can mostly be regarded as ad-hoc approaches in terms of visualization methodology, see the related work in Section 1.3. With this paper, we intend to

• present a novel solution to the problem that applies visualization technology optimizing layout and interaction,

• discuss our contribution in terms of visualization methods and how they relate to existing methods from other application areas, and

• show how our interactive visual exploration system is used in practice.

Instead of presenting yet another ad-hoc approach to visualize aligned biological networks, we built an interactive visualization system that allows for a systematic exploration of the data. Our system is based on a new 2.5D layout approach, see Section 2.1, and provides the user with various application-targeted interaction techniques to visually explore the alignment, see Section 2.2. The layout has to fulfill certain specific requirements, which are formulated in Section 1.4. How an application scientist can interactively and visually explore network alignments is described in an application scenario in Section 3.

### 1.2 Protein-Protein Interaction Networks and Key Questions

Protein-protein interactions (PPIs) are transient or permanent connections between proteins, and they are important for many biological phenomena such as signaling, transcriptional regulation, and multi-enzyme complexes. They are explained by molecular adhesive forces between parts of the proteins (domains) which in turn can be tracked down to the atomic level. The proteins of an organism and their interactions form a PPI network.

Interaction networks evolve by the loss and gain of nodes (proteins) and links (interactions). It is assumed that the complex networks interconnecting the components of an organism such as a human being are, like all of life, the result of a more or less gradual evolutionary process of descent with modification. Emergence of biological complexity is nevertheless poorly understood, and a deeper understanding is of utmost importance.

As the PPI networks of different species evolved from a common ancestor network, we are able to align them. A *network alignment *for a number of networks from different species specifies which nodes (representing the proteins) in one network correspond to (i.e. are orthologous to) which nodes in the other networks. This correspondence may be one to one, or it may relate a set of paralogs in one species to an orthologous set of paralogs in another species. More precisely, we view proteins from one species to be paralogous if they evolved by duplication after the speciation event splitting the last common ancestor. Two proteins in one species that evolved from the same protein are *not *understood as paralogous *if *they were already distinct proteins in the PPI network of the last common ancestor. In Additional File 1, we provide a more detailed discussion on the biological background of protein interaction network evolution. In a recent strand of research several groups have begun to systematically compare interaction networks between organisms, and the network of one organism with itself [3]. In the first case, orthologous subnetworks are inferred, as described above. Paralogous subnetworks can be detected in the second case. In particular, the PathBlast tool [6] can detect orthologous paths in two networks. Given a path or a small network to search for and a network to search in, it returns orthologs of the query path/network in the search network, displayed in a graphical "side by side" output [7,8]. PathBlast also aligns networks for more than two species. Another network alignment approach called "Local Graph Aligner" was developed based on a spin model [9]. This approach is used to align several networks and evaluates the statistical significance of the alignment. Yet another approach, NetworkBlast [10], uses an efficient representation of alignments and infers conserved complexes. The output of NetworkBlast can be used as input for VANLO. In another approach, networks are not directly aligned by their graph structure. Instead, they are aligned based on modeling the evolution of the networks from a common ancestral PPI network using Bayesian methodology [11]. This approach allows the alignment of more than two large networks. It does not only compute an alignment, it also explains how the networks evolved.

In biology, scientists are not only faced with PPI networks but with many other kinds of biological networks including regulatory ones that involve DNA-protein interaction and metabolic ones that include small metabolites as nodes. These networks are also related by evolution and can be aligned. Therefore visualization techniques developed for aligned PPI networks can also be used for these kinds of biological networks. Analysis of all kinds of networks will gain importance, in particular in biomedicine. After all, complex diseases must be tackled nowadays: cancer, arteriosclerosis and dementia are all multifactorial. They all have their cause in the interplay of a multitude of factors, many of which corresponding to networks gone out of order. In this context, comprehensive visualization can be a trigger of medical progress.

Given aligned PPI networks of different species, biologists are particularly keen on having means to answer the following questions:

• What is the conserved core of the alignment, i.e., its most ancestral part?

• What are the cores of the underlying pairwise alignments?

• What is new in each network?

The core of an alignment consisting of orthologous proteins in all species that share the same interactions most likely consists of proteins responsible for the same biological process and with the same function. This insight allows biologists to predict some protein properties from aligned PPI networks [4]. Furthermore, the core of an alignment is a good estimate for the network of the last common ancestor of the species involved. The pairwise cores are good estimates for the last common ancestor network of two species. Hence, they should be explored for the networks of two species that are close in the species tree. Detection of pairwise cores can help biologists to reconstruct the evolution of parts of the PPI network.

Newly developed parts in a PPI network are usually assumed to represent new functionality, that did not exist before. After being identified, this new part may afterwards be subject to further investigations. Network comparison should allow to find putative errors in one of the networks, or in the alignment. One hint for an error (mostly an error in the underlying databases) could be an edge existing only in one of the species, and the user can have a closer look, trying to find out what the evidence for this edge is and whether this interaction really exists.

### 1.3 Related Work

#### 1.3.1 Graph Drawing

It is intuitive to represent biological networks such as PPI networks as graphs. In a PPI network the proteins can be represented as vertices of a graph and the PPIs as edges of the graph. Therefore, visualizing biological networks is a special subject of graph drawing which is a well-studied field in information visualization [12].

The layout of a graph is most important because it determines the human perception of the graph [13]. In graph drawing one is generally interested in optimizing the layout of the graph with respect to some properties and constraints. Many different approaches exist, depending on the properties of the graph or on the information one is interested to visually extract or highlight. Graphs are most commonly drawn using a 2D layout where vertices are drawn as nodes and edges represented by lines. Plenty of algorithms exist for automated graph drawing [14]. Probably the most prominent approach to layout a graph is given by the family of force-directed algorithms [15-20]. The goal of these algorithms is to group interconnected nodes together and to spatially separate non-connected nodes. Therefore, attracting and repelling forces are defined and applied for node interference. Typically, all nodes repel each other using pairwise repelling forces and all connected nodes attract each other (up to a minimum distance). Algorithms like the one by Fruchterman and Reingold [18] or the one by Kamada and Kawai [19] iteratively compute a displacement for each node determined by the defined forces until convergence. The advantage of these algorithms is their flexibility, i. e. the possibility to define the forces according to a special application, which makes these algorithms suitable for many different graphs in diverse applications. Another iterative approach is to define an energy function which penalizes bad properties of the layout, and then to use simulated annealing or another optimization algorithm for iteratively optimizing this function [15]. Within the field of biology, a wide range of graph layout algorithms are considered as can be seen in the numerous visualization tools for biological networks like Cytoscape [21], ProViz [22], VisANT [23], or VANTED [24].

#### 1.3.2 Visualizing Aligned Networks

Aligned networks can be regarded as a set of graphs, where the alignment establishes connections between the graphs or, more precisely, between entities of the graphs (e.g., some of the nodes are aligned across the networks). For visualizing an alignment of PPI networks different approaches have been considered and are used today. For a detailed survey on the state of the art in visualizing aligned biological networks we refer to our report [25], where we divide the approaches into two main classes, namely "side by side" and "all in one".

The "side by side" approach, follows the idea to draw the individual aligned networks next to each other in a 2D layout and to highlight the aligned nodes by the same relative position and/or additional edges connecting them [3,6,26]. The advantage of this approach is that it is able to intuitively handle paralogous proteins. However, this approach is inappropriate for large network alignments and is hardly readable if there are many additional edges for representing the alignment relation.

The "all in one" approach draws the aligned networks in just one node-link diagram where one node represents the orthologous proteins of all networks [27,28]. Obviously, fewer edges and nodes are needed with this visualization but problems with the interpretation of the edges and also with displaying paralogs arise [25]. These problems can be alleviated to some degree by using the idea of metagraphs [29].

An appropriate solution that combines the advantages of both classes is given by using 2.5D layouts [30], where the individual networks are laid out in 2D and the relationship of the entities is implied by drawing all 2D layouts simultaneously using the third dimension and by placing corresponding entities on top of each other. Schreiber [31] used such an approach for the comparison of different biological networks in the context of metabolic pathways. However, his approach does not support the visualization of paralogous entities (proteins). Moreover, he did not provide any interactive exploration methods and his approach is specialized for metabolic pathways and a KEGG [32] like layout.

In terms of visualization methodology, visualizing aligned biological networks is related to the representation of evolving graphs. When considering evolving (dynamic) graphs one deals with one graph that changes over time, instead of an alignment of related graphs. Several approaches for so-called dynamic graph drawing exist [33-36]. The layout considerations of these approaches could easily be adopted to laying out aligned networks, where the split representation, i.e., each time step is shown in a separate drawing window, corresponds to the "side-by-side" layout and the merged representation, i.e., all time steps integrated into one drawing window, corresponds to the "all-in-one" layout. Some dynamic graph drawing approaches also consider a 2.5D approach with each time step drawn in a separate layer where the layers are placed on top of each other [37,38]. Given the key questions formulated in the section 1.2, we observed that they can be more intuitively answered when using our novel 2.5D layout algorithm, which considers the specific layout requirements described in Section 1.4. In particular, following these requirements, paralogs as well as orthologs can be identified easily.

### 1.4 Layout Requirements

For the visualization of aligned biological networks several approaches exist and they were surveyed and discussed in our report [25] where we derived some general layout requirements. We generally assume, as all existing approaches do, that the layout should be displayed as a node link diagram. Therefore, the general requirements for node link diagrams should be met also by a layout for aligned networks. Such general requirements are:

• All nodes should be clearly separated,

• nodes connected by an edge should be placed close to each other to prevent long edges,

• the number of edge crossings should be minimized, and

• available space should be used in an optimal way.

As a network alignment is not just a simple graph without further constraints. We derived some specific requirements that should be met by aligned network layouts. These specific requirements, designed to address the key questions outlined in Section 1.2, are:

• The structure of individual networks should be easily identifiable,

• individual networks should be clearly separated,

• alignment relations, i.e., which nodes and links are corresponding to which nodes and links in other networks, should be shown in a visually intuitive manner, and

• the core of the alignment should be easily retrievable and comprehensible.

## 2 Implementation

### 2.1 The Layout

We developed a novel interactive visual network exploration system with respect to the requirements specified above. Its main features are an appropriate aligned network layout and a range of helpful interaction mechanisms to visually explore the alignment.

#### 2.1.1 2.5D Setting

Taking into consideration the approaches discussed in Section 1.3, our layout is based on a 2.5D setting for the aligned graphs. The different networks are laid out in separate equidistant layers placed on top of each other.

To support an intuitive understanding of orthologous proteins of different networks, orthologs are assigned the same 2D position across the different layers. Therefore, the alignment relation is naturally and intuitively embedded into the layout and no additional edges, connecting the orthologous proteins, are required, as they are in "side by side". Thus, we only use one type of edge, namely the interaction edges between proteins, which keeps the visualization simple.

Paralogs are handled such that they are drawn closely together in a structured way at 2D positions within a well-defined area around the 2D position of the orthologous partners. Hence, paralogous structures can easily be identified.

#### 2.1.2 Strategy

For visualizing aligned networks with the above-mentioned layout representations ("side by side" and "all in one", or 2.5D setting), the networks are first laid out as node link diagrams in 2D. For the three layout representations the same layout algorithm can be applied, because all of them need the individual networks laid out in 2D with general graph drawing requirements and the orthologs of the different networks should have the same position.

To ensure this global layout structure, where the orthologous sets of paralogs of the different networks are positioned to the same 2D positions within the respective layers, the aligned networks need to be handled simultaneously. The strategy of our layout algorithm is

1. to build one common graph representing the complete network alignment by merging the corresponding orthologous sets of paralogs into one node,

2. to lay out this merged graph in 2D using known graph layout algorithms,

3. to split the previously merged paralogs and compute their local arrangement within each network, and

4. to map the networks to different layers, which are rendered in a 2.5D setting.

The first three steps are independent of the 2.5D setting such that other settings ("side by side" or "all in one") can be used, if desired.

#### 2.1.3 Layout Algorithm

**Figure 1:**
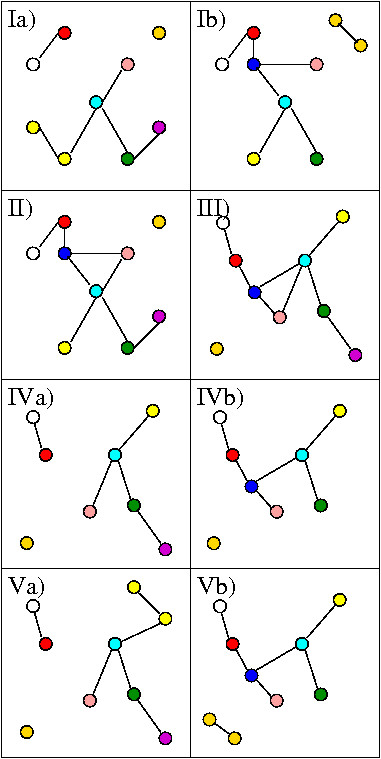
**The layout algorithm**. In this example proteins with the same color are orthologous (if they are in different panels in rows 1, 3, or 4 of the figure) or paralogous (if they are in the same panel). The two networks in Ia) and Ib) are first merged to the graph in II). This graph is now laid out, as seen in III). This layout is assigned to the two individual networks as shown in IVa) and IVb). In the last step paralogs, that are still merged in the individual networks are laid out and the results are shown in Va) and Vb).

Our algorithm consists of four steps, which are described in this section. In Figure 1, we illustrate the individual steps by giving an example. The example alignment consists of the two networks shown in Figure 1Ia) and 1Ib), where nodes with the same color are corresponding. Corresponding nodes are orthologs if they appear in different panels and they are paralogs if they appear in the same panel.

#### Merging into one graph

The given network alignment can be understood as one large graph with proteins as nodes. In a first step we collapse each orthologous set of corresponding paralogs, into one node. Hence, all proteins orthologous to each other are represented by a single node in this merged graph. All edges in the merged graph represent PPIs. The merged graph for our example is shown in Figure 1II). The advantage of using a merged graph is twofold. First, the orthologous proteins are already assigned to the same position, and secondly, the remaining graph is smaller and computing its layout becomes easier because the traditional layout algorithms usually work better on small graphs.

#### Computing the layout of the merged graph

The merged graph now is laid out in 2D by applying one of the graph layout algorithms mentioned in Section 1.3. For biological networks no additional graph-theoretical information such as planarity or density can be assumed a priori. Therefore, no special layout algorithm for graphs with certain properties can be used. Heuristic methods are a good choice in this case. In our visualization system we provide the use of two force-directed algorithms, namely the one by Fruchterman and Reingold [18] and the one by Kamada and Kawai [19]. In addition, we provide the use of a simulated-annealing algorithm [15], as it allows us to define an energy function adapted to our needs. The user may choose her/his preferred algorithm or she/he may simply test all three options and pick the result she/he likes best.

For our example the new layout is shown in Figure 1III).

In our simulated annealing approach we have four main terms. We sum up the lengths of the edges, the number of edge crossings, and the inverse of the angles between all pairs of incident edges to penalize these properties. We also add penalties if two nodes are too close to each other, in order to always clearly separate all nodes. If nodes consist of paralogous proteins, the lengths of their adjacent edges are divided by the number of paralogs to allow longer edges and therefore more space for these nodes.

#### Undo the merging step

Starting from the merged layout where all orthologous sets of paralogs have the same position, the final layout is computed. First the node positions computed for the merged graph are distributed onto the nodes of the individual networks, as shown in Figure 1IVa) and 1IVb). Afterwards the positions of the paralogous proteins have to be modified, because they still have the same position. These layout computations for the sets of paralogs can be done for each network individually. For one set of paralogs the free space around the position that is assigned to the set is determined according to the number of merged paralogs. Recall that the energy term used in the previous step allocates more space for merged paralogs. Within this free space local 2D arrangements for the small subgraphs of paralogs need to be determined. The local arrangement we chose for our implementation is to distribute the paralogs equidistantly on a small circle within the free space, where the center of the circle is the previously assigned 2D position. After this step, the layout of the layers is completed, see Figure 1Va) and 1IVb). In each of the networks there was just one set of paralogs to be laid out.

#### Assigning the 2.5D setting

From the graph layout the 2.5D representation of the aligned networks is obtained by assigning each network an individual layer displayed in Cartesian coordinates at equidistant heights *z*. For each node, a three-dimensional primitive is rendered at (*x*, *y*, *z*) where (*x*, *y*) are the coordinates computed by the algorithm and *z *is the assigned height for the network. The edges are connecting the nodes inside each individual network and therefore lie automatically in one layer, i.e. the start- and endpoint have the same height coordinate *z*. No edges between different layers are necessary, as orthologous groups are rendered on top of each other and are therefore easy to identify just by position.

### 2.2 Interactive Visual Exploration

The layout algorithm presented in the previous section generates an overall arrangement considering all proteins and all relations among them. When exploring the data, the user may be interested in seeing the entire structure, but typically also wants to concentrate on certain aspects. We provide interaction mechanisms that support such a visual exploration and analysis. Since all interactions operate on our 2.5D graph layout embedded in 3D space, all views are consistent and embedded into the overall context.

For the description of the interaction mechanisms that are supported by our system, we make use of the taxonomy introduced by Yi et al. [39].

#### Explore

Since we are using a 2.5D layout, rotation, translation, and zooming are supported. Different angles highlight different aspects of the data set.

#### Reconfigure

Although our 2.5D layout serves as the basis for all exploration tasks, we still support 2D layouts. One reason is that application scientists are currently used to look at 2D layouts. Providing the 2D layouts in addition to our 2.5D layout allows them to easily correlate our visualization to what they have in mind. We hope that this reduces the barrier to use our tool. Another reason is that 2D layouts may be beneficial for non-interactive visualizations which may be rendered for publications. We support both traditional 2D layouts, i.e. "side by side" and "all in one".

#### Encode

We support different color encodings for different networks. In addition, nodes can be encoded by shape information.

#### Abstract/Elaborate

When exploring the entire aligned network, showing all paralogs may hinder the comprehension of the global structure. Therefore we support an abstraction mechanism that collapses nodes representing paralogs into just one node. When investigating a certain substructure these paralogs are, of course, important to display therefore we can undo the abstraction at any time.

#### Filter

It is obvious that filtering is one of the main interaction features. In particular, we allow displaying/hiding edges or even complete individual networks. Of course, filter operations embed other interaction mechanisms like elaborating on paralogs.

In addition we found it useful to allow the user to store layouts for alignments to continue the exploration at a later time point, and to allow the user to take screen shots.

## 3 Results and discussion

For our application scenario we decided to use an alignment of the PPI networks of five species. We chose the PPI network of the insulin/IGF1 pathway. This pathway is of major importance not just in diabetes research, but it is relevant to molecular ageing in general [40]. The interaction data for our example is taken from the STRING [41] Web server (version 8.0), which integrates different kinds of biological data, for example databases such as KEGG [32], for building a protein interaction network. We integrated interactions traceable to databases or experiments; we did not use any data based on other evidence such as text-mining because they often contain errors. We only trusted interactions with a high confidence (STRING confidence score >0.7) and we deleted a few interactions that were listed by STRING under the label 'Experimental Data' even though they were predicted by orthology (e.g. the interaction between PI3K and IRS1 in Pan troglodytes has a score of 0.768 in STRING, but no experimental evidence).

**Figure 2:**
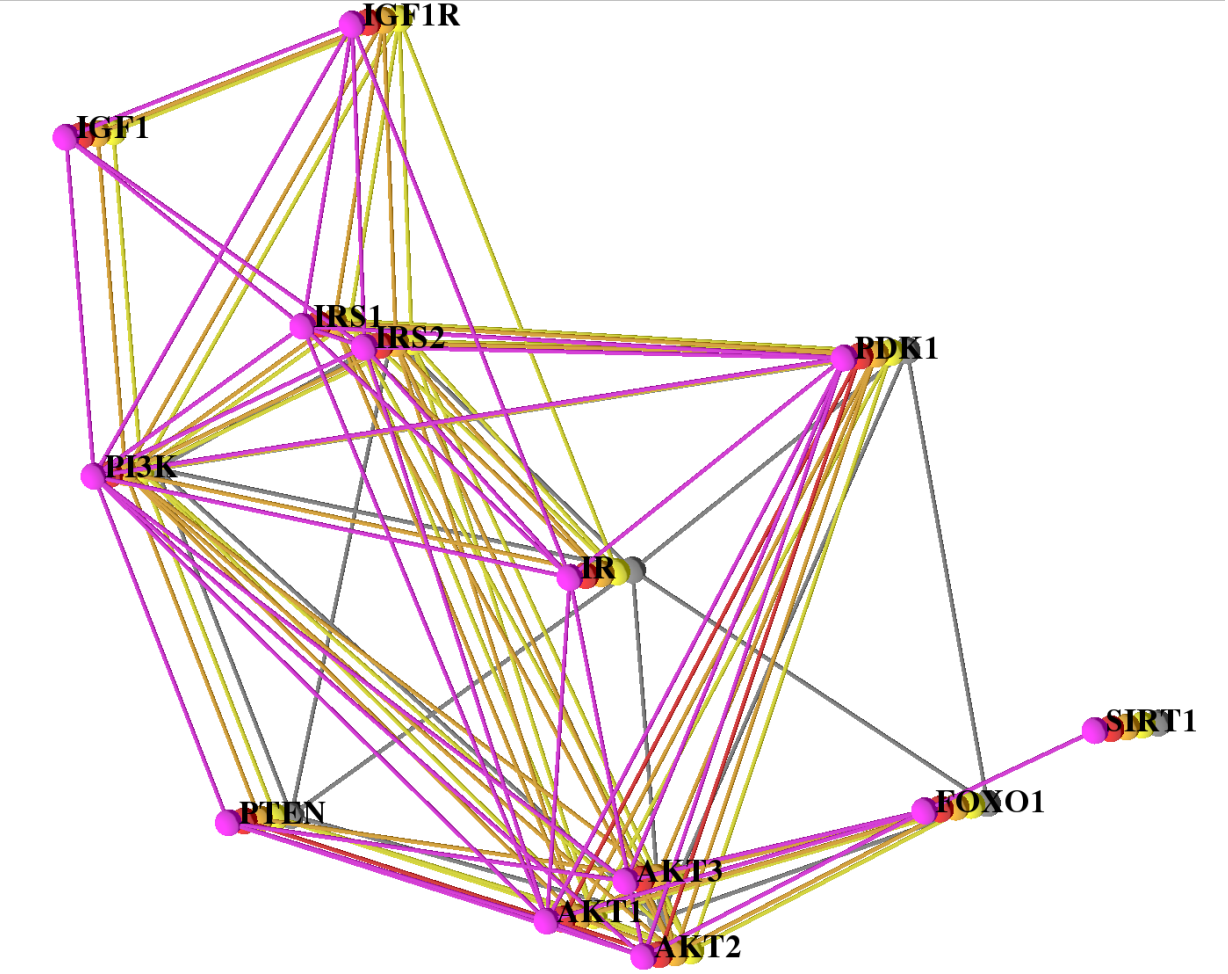
**The Insulin/IGF1 Pathway**. Alignment of human (pink), chimpanzee (red), mouse (orange), rat (yellow), and fly (gray). The layout is computed using our simulated annealing algorithm. All five species are shown and all paralogs (AKT1,2,3 and IRS1,2), too. For a better overview only the human network is labeled.

Finally, we manually investigated interactions scoring between 0.6 and 0.7 and added them, if STRING listed experimental evidence from BioGRID [42], BIND [43] or HPRD [44]. For the detection of synonyms and orthologs and also for the detection of paralogs we used iHop [45], HomoloGene [46], and Ensembl [47]. For the insulin/IGF1 network we found sufficient data for human, chimpanzee, mouse, rat, and fly. In the following we use our visualization system to explore the network alignment that is shown in Figure 2. The network of each species is shown in one layer and they are additionally color coded as follows: human (pink), chimpanzee (red), mouse (orange), rat (yellow), and fly (gray). Two aspects of network evolution and some artifacts due to missing data catch the eye immediately.

In the 2.5D layout in Figure 2, we can see that the IGF1/IGF1R part of the network (top right of the figure) is not found in fly (gray) but it exists in mammals, and we infer that it evolved in the lineage from the common ancestor of fly and mammals (called the ancestral bilaterian animal by zoologists, see ) to mammals. This observation is in concordance with Russell and Kahn ([40], Box 1). More data (on deuterostomic animals at the later branching points along the lineage from the bilaterian ancestor to mammals, such as sea urchin, sea squirt, lancelet, fish, frog, and/or bird) would enable us to set a more precise time point at which this part of the network may have evolved. The fly network (gray) is devoid of any paralogs; complexity of the pathway in mammals increased by duplication. The paralogs that evolved in the mammalian species form two clusters, the IRS cluster and the AKT cluster, and the visualization makes it clear that these two clusters of duplicated nodes are accompanied by a large number of duplicated edges. Tracking these down in STRING, we observe that the duplicated edges are derived from KEGG. However, KEGG does not describe the interactions of each paralog individually. Instead, it only lists the interactions of one representative AKT/IRS protein, and data processing by STRING was done under the assumption that the interactions are valid for each paralog, an assumption that is not necessarily true. Thus, the duplicated edges may be a data processing artifact. On the other hand, if the assumption is true, the interpretation is that in the insulin signaling pathway, interactions were usually kept after gene duplication leading to paralogs. For example, the number of edges from PI3K to the IRS cluster equals the number of IRS paralogs (two for human, mouse and rat and one for fly, see also Figure 2) except for chimp, where for PI3K there is no interaction with the other proteins, as discussed below. Such a scenario, if it reflects biological reality and is not a database artifact, indicates that the IRS paralogs are alternative stopovers in the standard signaling chain from IR to PI3K, via IRS (see [40], Box 1), indicating redundancy. (One specific explanation comes to mind: interaction data are often pooled over tissue types, so that it may well be that alternative paths are employed in different tissues, and these are regulated in a tissue-specific way.)

**Figure 3:**
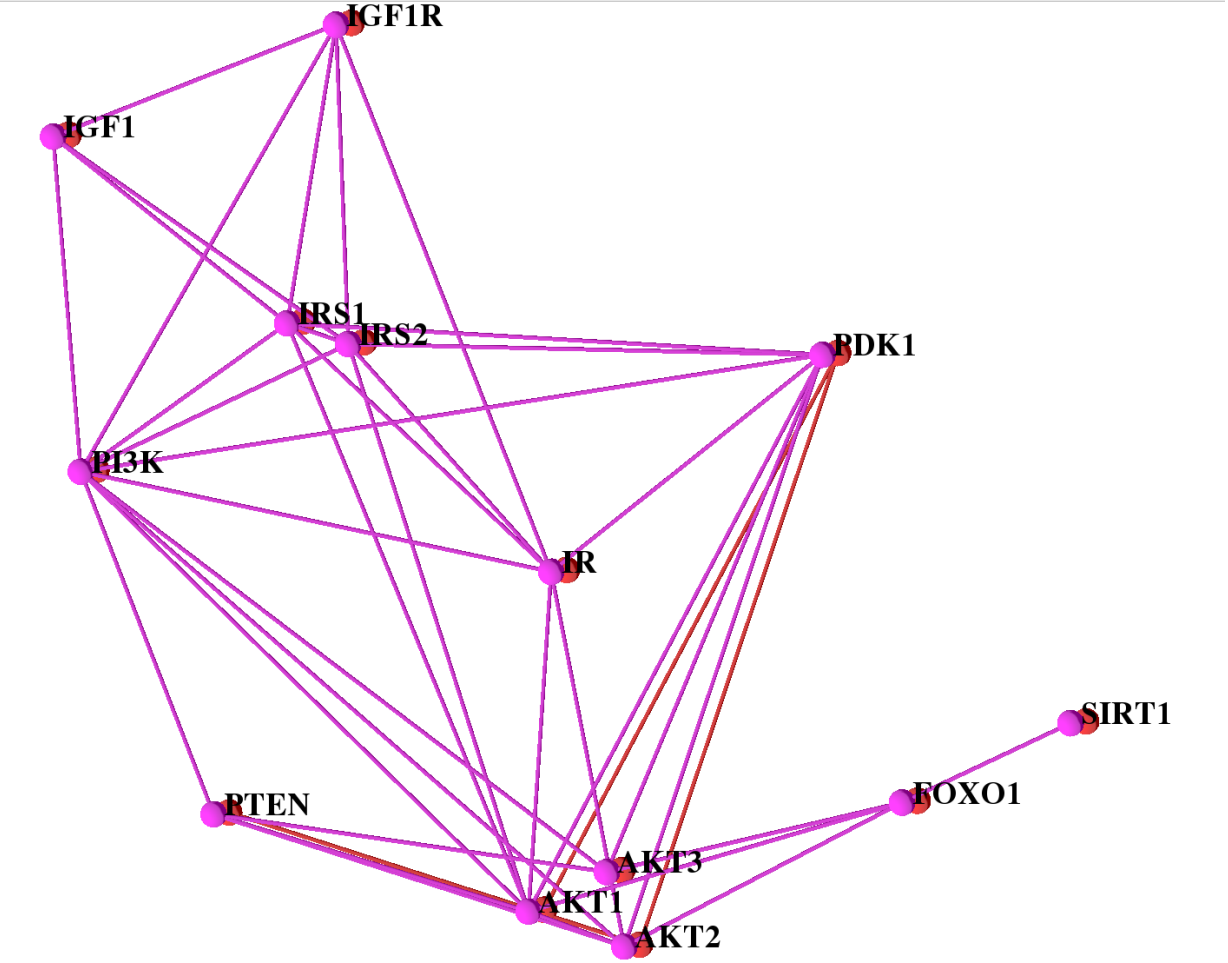
**The Insulin/IGF1 Pathway alignment of human (pink) and chimpanzee (red)**. The other networks are filtered out. For a better overview only the human network is labeled.

Looking at the red network (chimpanzee, or chimp for short), a large number of interactions (edges) existing in the other networks are missing. In this situation filtering out the other networks and only looking at the network of the chimp and the human one for comparison supports the exploration. This is done easily and the filtered view is shown in Figure 3. In chimp, only PDK1, PTEN and AKT are connected. There are no links (no red edges) connecting PDK1, PTEN and AKT to the other proteins. Here, the biologist interpreting the network must know two facts for a correct analysis: (a) human and chimpanzee are very closely related; their genomes and physiology are very similar, and (b) mouse and rat together form a group that is in turn related to the human/chimpanzee group. Thus, the biologist concludes that the missing edges must be due to missing data in STRING, and that they are not yielding insight into network evolution. In fact, chimpanzee data are just recently becoming available and it is no wonder that these are incomplete. Moreover, the biologist can use the network alignment to predict missing components (nodes and/or edges) in the chimpanzee network which is expected to be almost identical to the human one.

The interaction of fly FOXO1 (also known as dFOXO, Afx or CG3143) and IR (Figure 2 center) is only displayed in case of fly. Tracking down the link in STRING, an entry from the BIND database [48] is listed as evidence, which in turn cites Puig et al. [49]. Their abstract includes the sentence "dFOXO [...] activates two key players of the dInR/dPI3K/dAkt pathway: the translational regulator d4EBP and the dInR itself". In short, FOXO activates InR in fly, where InR (Insulin receptor) is the ortholog of IR (Insulin receptor) in mammals. It is possible that the feedback loop IR → PI3K → AKT → FOXO → IR (see also [40], Box 1) is not just active in fly, and that it also exists in the other species. Here, our visualization yielded an interesting hypothesis, which is not so obvious in a series of "side by side" renderings.

**Figure 4:**
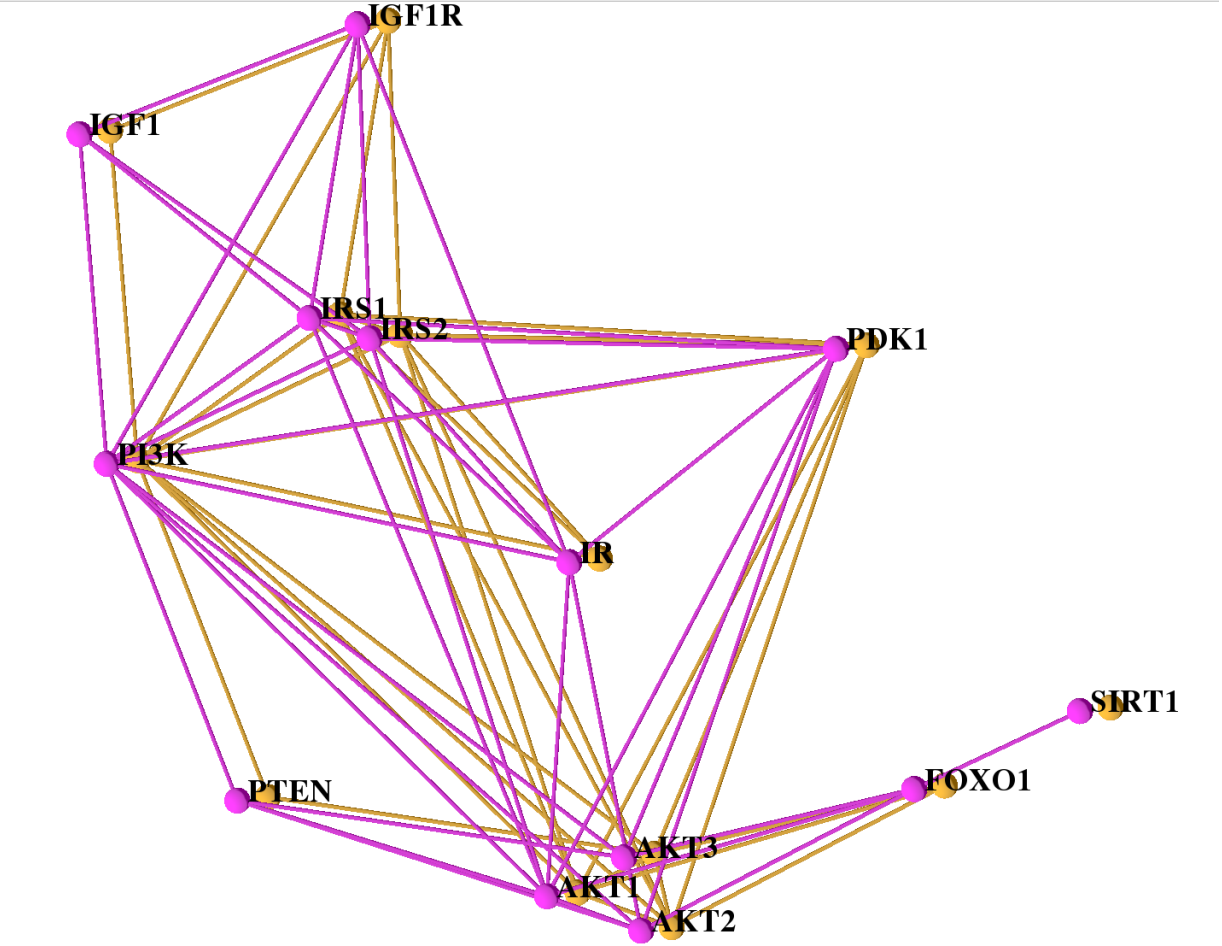
**The Insulin/IGF1 pathway alignment of the network for human (pink) and mouse (orange) only**. The same layout and settings as in Figure 2 are used but the other three species are filtered out. Both networks are rather similar, the few differences (e.g. missing interaction between SIRT and FOXO1 in mouse) are easily recognizable.

Using filtering operations to mask out chimp, rat, and fly allows an easy comparison of human and mouse as shown in Figure 4. In this Figure the eye can easily identify the identities and the differences. First of all, there is no difference with respect to the nodes. However, some links in human are missing in mouse. For example, these are links from SIRT1 to FOXO1, from IR to IGF1R, and from IGF to IRS. All these links can be traced back to human-specific data incorporated into STRING; the links are reported in a publication supporting a BIND entry [50] or they are derived from HPRD [44] and PID [51].

Finally, with the help of our visualization we are able to identify the core of the network alignment, which consists of the nodes and edges that are present for the largest number of species. Setting the minimum species threshold to 2, the core does not include the link between FOXO and INSR (only present in fly) that we discussed above, nor the interactions FOXO1 → PDK1, IRS → PTEN and PTEN → IR (in fly), nor the interactions that are present only in human.

**Figure 5:**
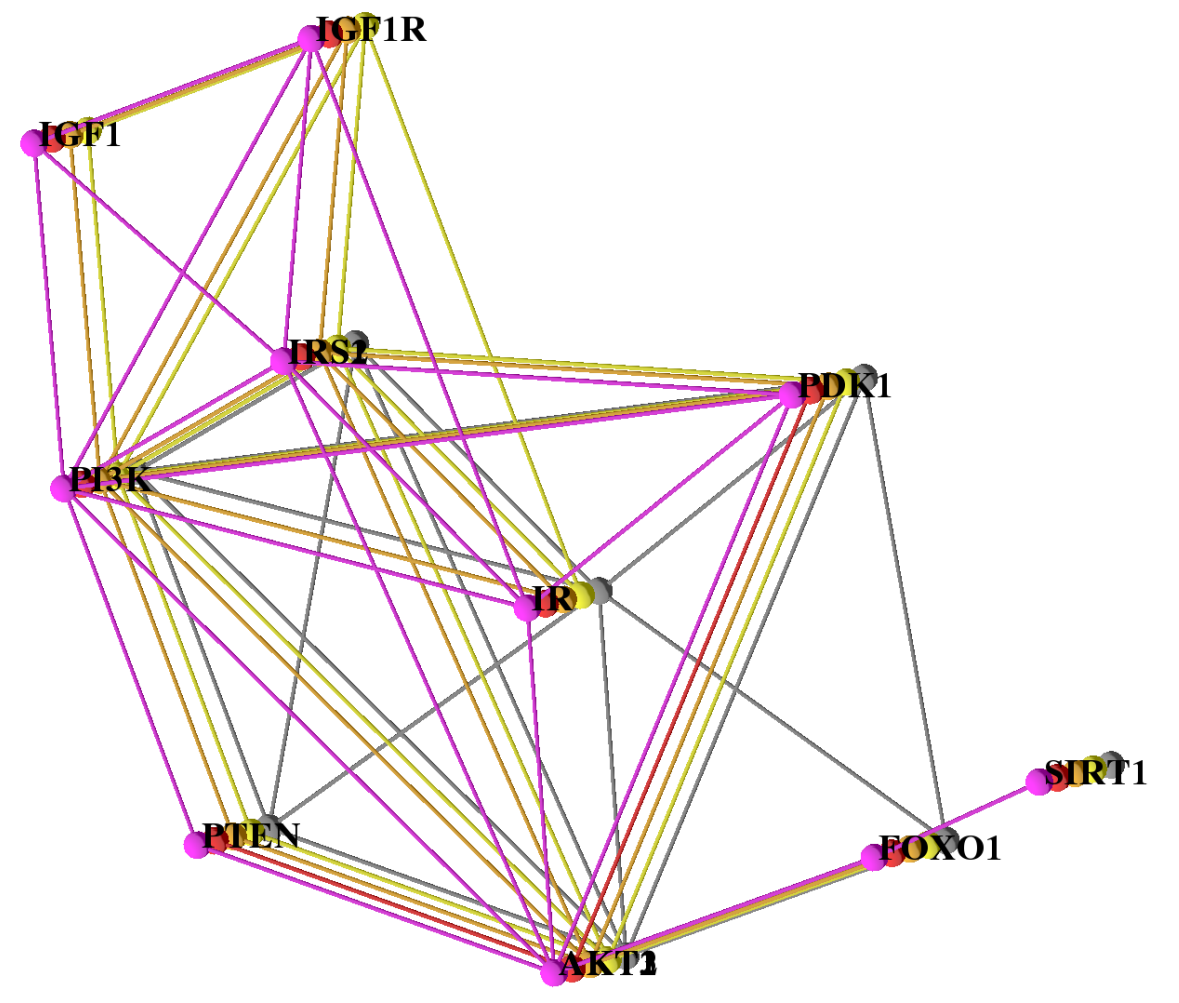
**A network alignment where all paralogous nodes have been collapsed**. The alignment features the Insulin/IGF1 Pathway as in Figure 2, with the same layout. Fewer interactions and fewer proteins are shown, yielding a better overview of the overall structure.

If there are many paralogs it is very useful to use abstraction, by collapsing the paralogs, reducing the number of displayed nodes and edges. The information for finding the core network will nevertheless not be missing in this abstract view, see Figure 5. In particular, the edges from PI3K to IRS can be seen much easier in Figure 5 than in Figure 2.

**Figure 6:**
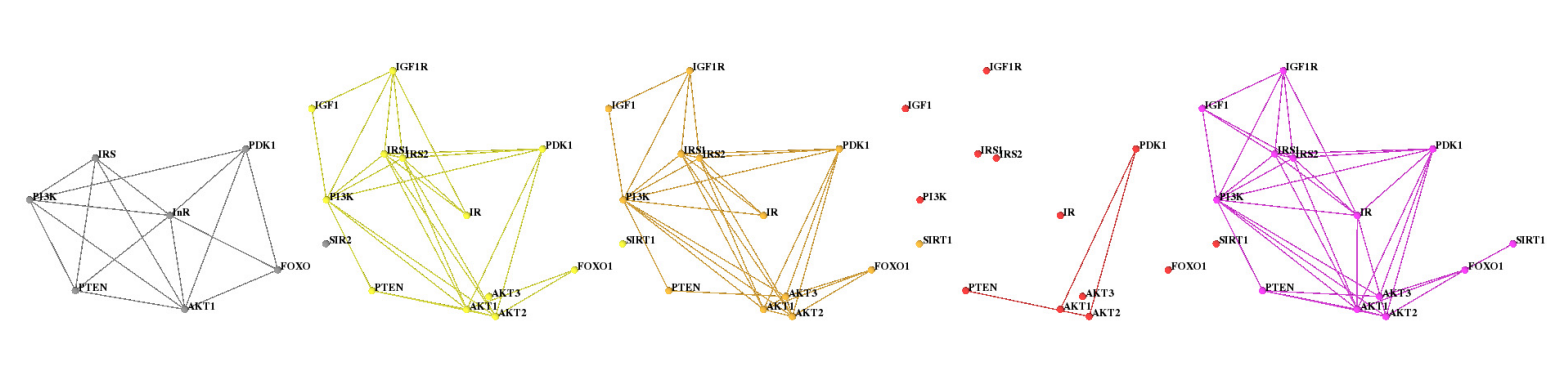
**The Insulin/IGF1 pathway alignment of the network for all five species, human (pink), chimpanzee (red), mouse (orange), rat (yellow), and fly (gray) in a "side by side" setting**. The same layout as in Figure 2 is used.

Researchers interested in one of the traditional layout settings such as a "side by side" layout, can obtain one by a mouse click, see Figure 6. In this setting one can easily see that there are many edges missing in the network of the chimpanzee (green). However, it is hard to recognize which edges do exist in most of the networks and therefore might belong to the core of the alignment. Moreover, it is hard to recognize the novel interactions discussed above.

**Figure 7:**
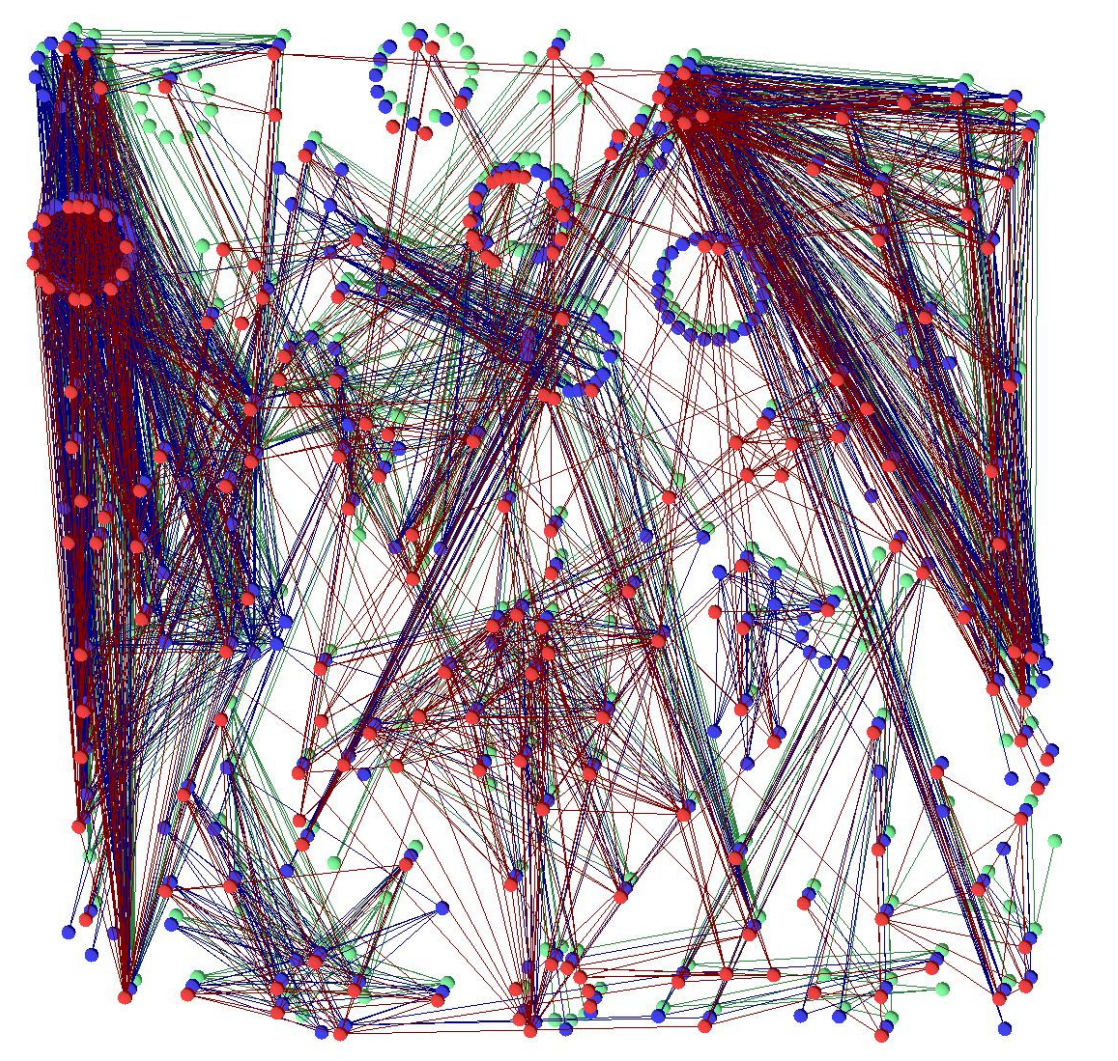
**A large alignment with more than 200 proteins per species and nearly 800 nodes overall**. The layout for this alignment is computed by our simulated annealing algorithm in less than 30 seconds.

**Figure 8:**
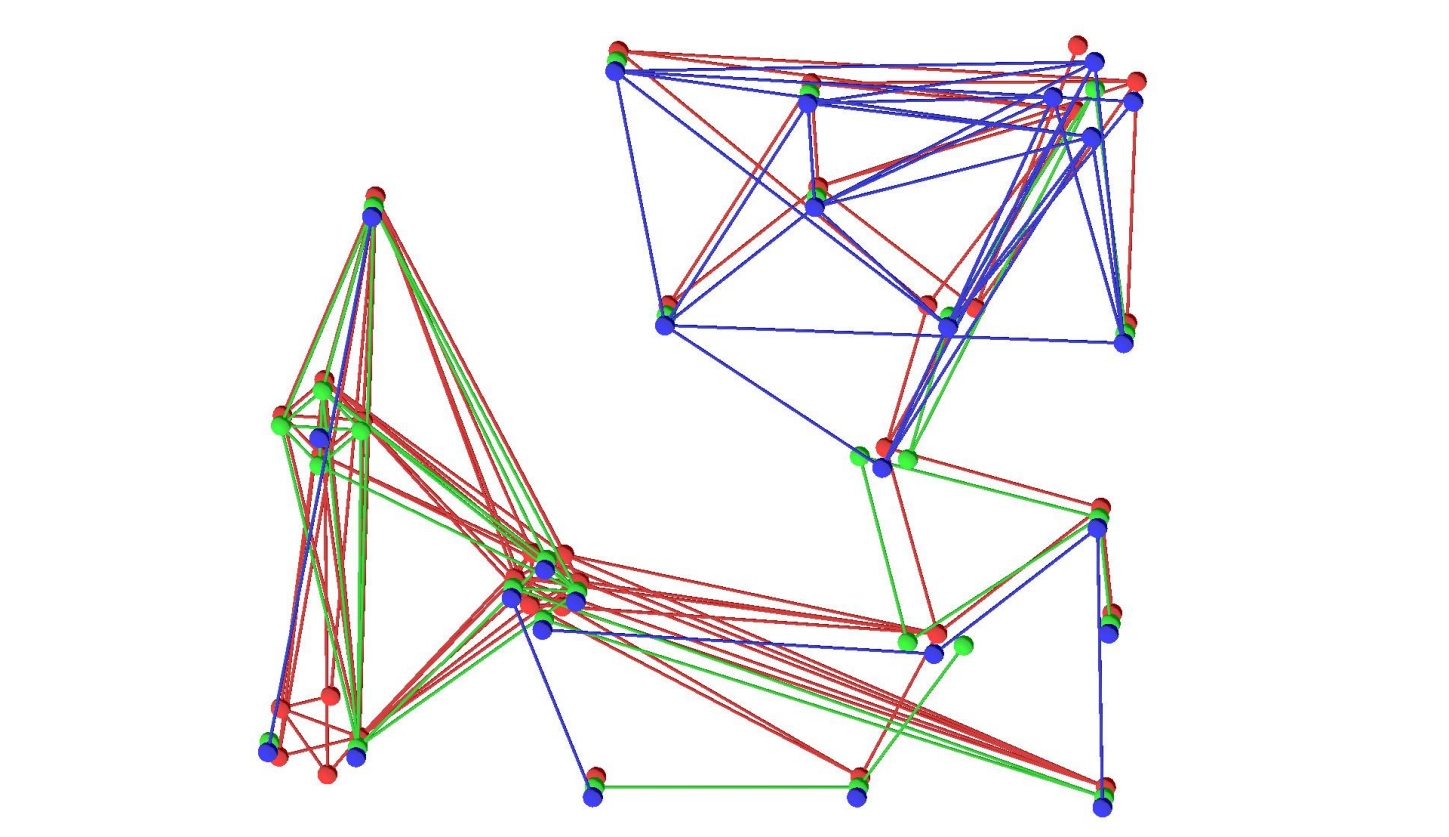
**A sparse network alignment for three species**. In the part on the left, most of the blue network is missing and in the part on the right, most of the green network is missing.

The example shown up to now is rather small, five networks with around ten proteins each, altogether around 60 proteins. But VANLO is able to handle larger network alignments with hundreds of proteins too. An example dataset with three networks and a total of nearly 800 proteins can be navigated interactively and a layout with our simulated annealing algorithm was computed within less than 30 seconds, see Figure 7. Another visualization challenge is a sparse alignment, with a small overlap between the different species, see Figure 8. The collapsed graph of an alignment (see Section 2.1.3) does not depend on the number of networks in which there are orthologous proteins and therefore the layout computation for sparse graphs is as efficient as the one for dense graphs.

In conclusion, our tool can be used for the detailed inspection of the similarities and differences of alignable interaction networks, as we did for two (human and mouse, Figure 4) and five networks (Figure 2). In turn, a bird's eye view of the latter alignment provided by our tool yielded some quick insights into regions where paralogs are abundant, and regions where some subnetworks are not represented. Interaction mechanisms supported the analysis tasks by filtering the required information and facilitating an interactive display of the parts to be investigated.

## 4 Conclusion

The visualization system for aligned biological networks (VANLO) we presented, enables the user to answer some key questions concerning network alignments. It also provides several interaction techniques allowing the user to visually explore aligned networks. Additionally, a new layout approach using 2.5D is presented. This approach fulfills all requirements for a layout of alignments. The layout turns out to be helpful to understand the structure of a network alignment. Also, traditional representations are supported. Thus the visualization system is a very useful tool for biologists to explore alignments, to find out details and to render results.

With respect to limitations of the software and future work, it would be useful to automatically include properties of the proteins and to automatically map them to shape or color attributes. This would help the user to easily predict properties of proteins, where they are not known. Regarding the edges, it would be useful to allow different edge/arrow shapes, for example, to denote regulation of a protein (gene product) by another protein (transcription factor). Moreover, for very large networks in particular (more than several hundred nodes), we are developing ways to transform/simplify these before rendering them, based for example on the ideas of Royer et al. [52]. Finally, a visualization of the entire evolutionary history of an aligned set of networks, starting from a small ancestral network, is on our agenda.

## 5 Availability and requirements

The software project presented in this manuscript is called VANLO (Visualization of Aligned Networks with Layout Optimization) and is available on . The presented software is implemented in C++, where the included graphs are implemented using the boost graph library and for the graphical user interface QT was used. The simulated annealing layout algorithm is an own implementation and the other layout algorithms are, sometimes modified, the ones provided by the boost graph library. This first publication of the software is only for the use with Windows XP but it will later on be published in a platform independent version. A manual for the software, including a file format description for the alignment data, and an explanation of the usage is given in Additional file 2. The work is currently published under the lesser gnu public license (LGPL), which allows every user to freely use the software.

## 6 Authors' contributions

SB did the implementation work and together with LL accomplished the theoretical work on the visualization ideas. GF developed the visualization scenario together with the interpretation and initiated this project. All three authors contributed to the manuscript. All authors read and approved the final manuscript.

## 7 Authors' Information

SB studied mathematics and recieved his Diploma in 2005 at the Ernst-Moritz-Arndt-Universität Greifswald, Germany. Thereafter he worked as a scientific member in the field of visualization and computer graphics at the Universität Greifswald, Germany, where he is actually doing his Ph.D. on visualization of protein interaction data. His research interests are in the fields of visualization and graph theory.

LL is an Associate Professor of Computational Science and Computer Science at the School of Engineering and Science of the Jacobs University, Bremen, Germany. He received his academic degrees from the Universität Karlsruhe (TH), Germany, including a Diploma in computer science in 1997 and a Ph.D. in computer science in 2001. He spent three years as a post-doctoral researcher and lecturer at the Institute for Data Analysis and Visualization (IDAV) and the Department of Computer Science of the University of California, Davis, U.S.A. He joined the Department of Mathematics and Computer Science of the Ernst-Moritz-Arndt-Universität Greifswald, Germany, as an assistant professor in 2004. Since 2006 he holds his current position at Jacobs University. LL's research interests are mainly in the areas of scientific and information visualization but include certain topics in computer graphics and geometric modeling.

## Supplementary Material

Additional file 1 **Background on Protein Protein Interaction Network Evolution**. In the second supplement, the file protein_background.pdf, the evolution of protein interaction networks of different species from one common ancestor species is explained. Due to their evolution from a common ancestor, PPI networks can be aligned. How an alignment is defined, is also explained in this supplement. Furthermore the reader finds a detailed explanation on orthologous and paralogous proteins. Click here for file

Additional file 2 **Manual**. The file manual.pdf includes a manual for the use of the VANLO software and a file format specification for the input files used. Click here for file

## References

[B1] Davidson EH, Erwin DH (2006). Gene regulatory networks and the evolution of animal body plans. Science.

[B2] Sharan R, Ulitsky I, Shamir R (2007). Network-based prediction of protein function. Mol Syst Biol.

[B3] Sharan R, Ideker T (2006). Modeling cellular machinery through biological network comparison. Nature Biotechnology.

[B4] Berg J, Lässig M (2006). Cross-species analysis of biological networks by Bayesian alignment. Proc Natl Acad Sci USA.

[B5] Boiani M, Schöler HR (2005). Developmental cell biology: Regulatory networks in embryo-derived pluripotent stem cells. Nature Reviews Molecular Cell Biology.

[B6] Kelley BP, Sharan R, Karp RM, Sittler T, Root DE, Stockwell BR, Ideker T (2003). Conserved pathways within bacteria and yeast as revealed by global protein network alignment. Proc Natl Acad Sci USA.

[B7] Kelley BP, Yuan B, Lewitter F, Sharan R, Stockwell BR, Ideker T (2004). PathBLAST: a tool for alignment of protein interaction networks. Nucleic Acids Res.

[B8] Sharan R, Suthram S, Kelley R, Kuhn T, McCuine S, Uetz P, Sittler T, Karp R, Ideker T (2005). Conserved patterns of protein interaction in multiple species. Proc Natl Acad Sci USA.

[B9] Berg J, Lässig M (2004). Local graph alignment and motif search in biological networks. Proc Natl Acad Sci USA.

[B10] Kalaev M, Bafna V, Sharan R, Vingron M, Wong L (2008). Fast and Accurate Alignment of Multiple Protein Networks. RECOMB, of Lecture Notes in Computer Science.

[B11] Dutkowski J, Tiuryn J (2007). Identification of functional modules from conserved ancestral protein protein interactions. Bioinformatics.

[B12] Herman I, Melançon G, Marshall MS (2000). Graph Visualization and Navigation in Information Visualization: A Survey. IEEE Transactions on Visualization and Computer Graphics.

[B13] Blythe J, McGrath C, Krackhardt D, Brandenburg FJ (1996). The Effect of Graph Layout on Inference from Social Network Data. Graph Drawing, Passau, Germany, September 20-22, 1995.

[B14] Di Battista G, Eades P, Tamassia R, Tollis IG (1994). Algorithms for Drawing Graphs: An Annotated Bibliography. Comput Geometry: Theory Appl.

[B15] Davidson R, Harel D (1996). Drawing graphs nicely using simulated annealing. ACM Transactions on Graphics.

[B16] Eades P (1984). A Heuristic for Graph Drawing. Congressus Numerantium.

[B17] Frick A, Ludwig A, Mehldau H, Tamassia R, Tollis IG (1994). A Fast Adaptive Layout Algorithm for Undirected Graphs. Proc DIMACS Int Work Graph Drawing, GD, 894.

[B18] Fruchterman TMJ, Reingold EM (1991). Graph Drawing by Force-directed Placement. Software - Practice and Experience.

[B19] Kamada T, Kawai S (1989). An algorithm for drawing general undirected graphs. Inf Process Lett.

[B20] Noack A (2003). An energy model for visual graph clustering. Proceedings of the 11th International Symposium on Graph Drawing (GD 2003), LNCS 2912.

[B21] Shannon P, Markiel A, Ozier O, Baliga NS, Wang JT, Ramage D, Amin N, Schwikowski B, Ideker T (2003). Cytoscape: a software environment for integrated models of biomolecular interaction networks. Genome Res.

[B22] Iragne F, Nikolski M, Mathieu B, Auber D, Sherman D (2005). ProViz: protein interaction visualization and exploration. Bioinformatics.

[B23] Hu Z, Mellor J, Wu J, Delisi C (2004). VisANT: an online visualization and analysis tool for biological interaction data. BMC Bioinformatics.

[B24] Junker BH, Klukas C, Schreiber F (2006). VANTED: A system for advanced data analysis and visualization in the context of biological networks. BMC Bioinformatics.

[B25] Brasch S, Linsen L, Fuellen G, Wolter FE, Sourin A (2007). Visualization of Aligned Biological Networks: A Survey. Proc 2007 International Conference on Cyberworlds.

[B26] Koyutürk M, Kim Y, Subramaniam S, Szpankowski W, Grama A (2006). Detecting conserved interaction patterns in biological networks. J Comput Biol.

[B27] Bandyopadhyay S, Sharan R, Ideker T (2006). Systematic identification of functional orthologs based on protein network comparison. Genome Res.

[B28] Hirsh E, Sharan R (2007). Identification of conserved protein complexes based on a model of protein network evolution. Bioinformatics.

[B29] Hu Z, Mellor J, Wu J, Kanehisa M, Stuart JM, Delisi C (2007). Towards zoomable multidimensional maps of the cell. Nature Biotechnology.

[B30] Brandes U, Dwyer T, Schreiber F (2004). Visual Understanding of Metabolic Pathways Across Organisms Using Layout in Two and a Half Dimensions. Journal of Integrative Bioinformatics.

[B31] Schreiber F (2003). Visual comparison of metabolic pathways. J Vis Lang Comput.

[B32] Kanehisa M, Goto S (2000). KEGG: Kyoto Encyclopedia of Genes and Genomes. Nucleic Acids Res.

[B33] Branke J, Kaufmann M, Wagner D (2001). Dynamic graph drawing. Graph Drawing - Models and Algorithms.

[B34] Brandes U, Wagner D (1997). A Bayesian Paradigm for Dynamic Graph Layout. GD '97: Proceedings of the 5th International Symposium on Graph Drawing.

[B35] Diehl S, Görg C (2002). Graphs, They Are Changing. GD '02: Revised Papers from the 10th International Symposium on Graph Drawing.

[B36] Görg C, Birke P, Pohl M, Diehl S (2004). Dynamic Graph Drawing of Sequences of Orthogonal and Hierarchical Graphs. Graph Drawing.

[B37] Erten C, Kobourov SG, Le V, Navabi A (2005). Simultaneous Graph Drawing: Layout Algorithms and Visualization Schemes. J Graph Algorithms Appl.

[B38] Brandes U, Corman SR (2003). Visual unrolling of network evolution and the analysis of dynamic discourse. Information Visualization.

[B39] Yi JS, Kang Ya, Stasko J, Jacko J (2007). Toward a Deeper Understanding of the Role of Interaction in Information Visualization. IEEE Transactions on Visualization and Computer Graphics.

[B40] Russell SJ, Kahn CR (2007). Endocrine regulation of ageing. Nat Rev Mol Cell Biol.

[B41] von Mering C, Jensen LJ, Kuhn M, Chaffron S, Doerks T, Krüger B, Snel B, Bork P (2007). STRING 7-recent developments in the integration and prediction of protein interactions. Nucleic Acids Res.

[B42] Stark C, Breitkreutz BJ, Reguly T, Boucher L, Breitkreutz A, Tyers M (2006). BioGRID: a general repository for interaction datasets. Nucleic Acids Res.

[B43] Bader GD, Betel D, Hogue CW (2003). BIND: the Biomolecular Interaction Network Database. Nucleic Acids Res.

[B44] Mishra GR, Suresh M, Kumaran K, Kannabiran N, Suresh S, Bala P, Shivakumar K, Anuradha N, Reddy R, Raghavan TM, Menon S, Hanumanthu G, Gupta M, Upendran S, Gupta S, Mahesh M, Jacob B, Mathew P, Chatterjee P, Arun KS, Sharma S, Chandrika KN, Deshpande N, Palvankar K, Raghavnath R, Krishnakanth R, Karathia H, Rekha B, Nayak R, Vishnupriya G, Kumar HG, Nagini M, Kumar GS, Jose R, Deepthi P, Mohan SS, Gandhi TK, Harsha HC, Deshpande KS, Sarker M, Prasad TS, Pandey A (2006). Human protein reference database-2006 update. Nucleic acids research.

[B45] Hoffmann R, Valencia A (2005). Implementing the iHOP concept for navigation of biomedical literature. Bioinformatics.

[B46] Wheeler DL, Barrett T, Benson DA, Bryant SH, Canese K, Chetvernin V, Church DM, DiCuccio M, Edgar R, Federhen S, Geer LY, Helmberg W, Kapustin Y, Kenton DL, Khovayko O, Lipman DJ, Madden TL, Maglott DR, Ostell J, Pruitt KD, Schuler GD, Schriml LM, Sequeira E, Sherry ST, Sirotkin K, Souvorov A, Starchenko G, Suzek TO, Tatusov RL, Tatusova TA, Wagner L, Yaschenko E (2006). Database resources of the National Center for Biotechnology Information. Nucleic Acids Research.

[B47] Flicek P, Aken BL, Beal K, Ballester B, Caccamo M, Chen Y, Clarke L, Coates G, Cunningham F, Cutts T, Down T, Dyer SC, Eyre T, Fitzgerald S, Fernandez-Banet J, Graf S, Haider S, Hammond M, Holland R, Howe KL, Howe K, Johnson N, Jenkinson A, Kahari A, Keefe D, Kokocinski F, Kulesha E, Lawson D, Longden I, Megy K, Meidl P, Overduin B, Parker A, Pritchard B, Prlic A, Rice S, Rios D, Schuster M, Sealy I, Slater G, Smedley D, Spudich G, Trevanion S, Vilella AJ, Vogel J, White S, Wood M, Birney E, Cox T, Curwen V, Durbin R, Fernandez-Suarez XM, Herrero J, Hubbard TJP, Kasprzyk A, Proctor G, Smith J, Ureta-Vidal A, Searle S (2008). Ensembl 2008. Nucl Acids Res.

[B48] Bader GD, Donaldson I, Wolting C, Ouellette BFF, Pawson T, Hogue CWV (2001). BIND-The Biomolecular Interaction Network Database. Nucl Acids Res.

[B49] Puig O, Marr MT, Ruhf ML, Tjian R (2003). Control of cell number by Drosophila FOXO: downstream and feedback regulation of the insulin receptor pathway. Genes Dev.

[B50] Yang Y, Hou H, Haller EM, Nicosia SV, Ba W (2005). Suppression of FOXO1 activity by FHL2 through SIRT1-mediated deacetylation. The EMBO Journal.

[B51] National Cancer Institute Center for Bioinformatics (2005). Pathway Interaction Database. http://pid.nci.nih.gov.

[B52] Royer L, Reimann M, Andreopoulos B, Schroeder M (2008). Unraveling Protein Networks with Power Graph Analysis. PLoS Comput Biol.

