# Supplementary material for: VANLO - Interactive visual exploration of aligned biological networks
Source: BMC Bioinformatics. 2009 Oct 12;10:327. doi: 10.1186/1471-2105-10-327 (PMC2766391; doi:10.1186/1471-2105-10-327)
Supplement: Additional file 1 — Background on Protein Protein Interaction Network Evolution. In the second supplement, the file protein_background.pdf, the evolution of protein interaction networks of different species from one common ancestor species is explained. Due to their evolution from a common ancestor, PPI networks can be aligned. How an alignment is defined, is also explained in this supplement. Furthermore the reader finds a detailed explanation on orthologous and paralogous proteins. [file 1471-2105-10-327-S1.PDF]

Steffen Brasch, Lars Linsen, Georg Fuellen

1 VANLO (Visualization of Aligned Networks with Layout Optimization) - Manual

1.1 Update and News

Newest instructions and software can be found at `http://www.math-inf.uni-greifswald.de/VANLO`.

1.2 Installation

The downloaded files include a file VANLO.exe, which can be executed in Windows XP / Vista. Please leave the .dll files in the same directory like the VANLO.exe as this file needs them. Alternatively the .dll files can be copied into your system directory (usually ...\system32\).

1.3 File Format Specification

The simple interaction format (sif) is a well known file format for PPI networks. It is for example used in Cytoscape [1] for the exchange of network data. As the name already indicates, this format is very simple and allows only the storage of the network without any additional information. This file format was adapted for the use with data of aligned networks, but it did not allow to have proteins in one species without any orthologous proteins in the other species. As we found this to be a problem, we changed the format to allow this case by introducing dummy protein names such as 'none1'. Nevertheless the new format is compatible with the old one, such that the data found in the literature (e.g. `http://www.cellcircuits.org/Sharan2005/`) can be visualized with our software as well as the output of NetworkBlast [2].

The files are textbased and therefore easily readable with any editor and can be manipulated this way. Each line consists of three columns, the first column are the source nodes, the second specifies the type of edges, and the third are the target nodes. We use the terms source and target only for the explanation, the edges are understood undirected. The first and the third column for an alignment of n species are build as follows:

```
prot1|prot2|...|protn
```

where prot$i$ is a protein in the i-th network and it is orthologous to all other proteins in this list. The second column contains n digits taken from the set $\{0, 1, 2, 3\}$, where the i-th number describes the interaction between the i-th protein in the first block and the i-th

protein in the third block. The numbers 1, 2 denote that there is an edge and 0, 3 denote there is no edge. The distinction between 1 and 2 as well as 0 and 3 from PathBlast [3] are not used in VANLO. The columns are separated by the space character.

As already mentioned we have extended the specification to allow proteins which do not have an ortholog in all other species. Therefore, in the blocks with the protein names one simply uses none% as name for a protein that does not exist.

A small example of a .sif file for two aligned networks is shown in the following and is visualized in Figure 1.

```
a|a 11 b|b
b|b 13 b'|b
a|a 13 c|c
b|b 31 c|c
a|a 31 none1|d
b|b 31 none2|d
```

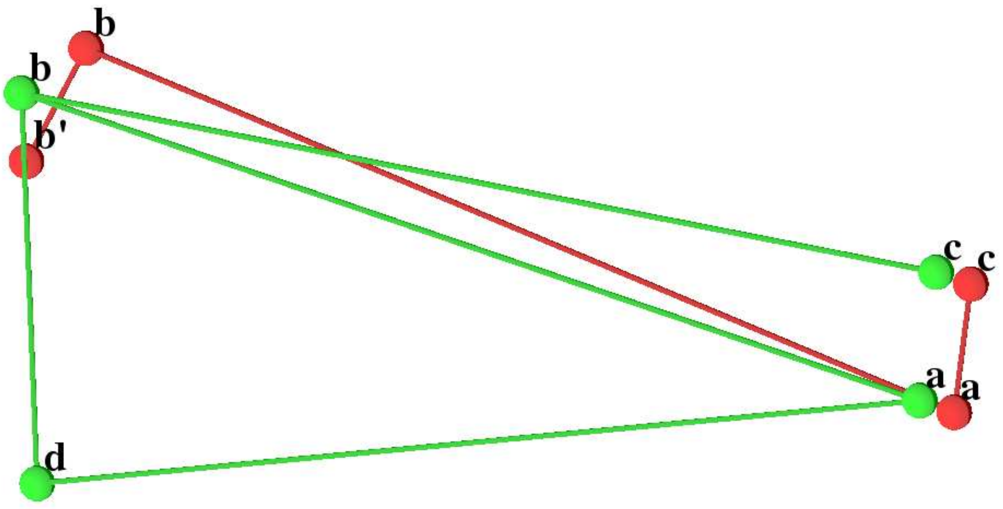

Figure 1: A small example for an alignment of two species. The first species is rendered in red and the second one in green. Note that the protein d in the second species has no orthologous protein in the first species.

1.4 Usage

The program has got two main modes. The first is the *layout computation mode* and the second is the *exploration mode*. The current mode is always printed at the right end of the menu bar. The first mode enables the user to compute a layout for the alignment, whereas the second mode does not allow any changes in the layout, therefore it allows to move around and view the alignment from different angles. Changing between the two modes is done in the menu *Mode*.

3D Navigation

In *exploration mode* the user has got different interaction possibilities explained below.

- Rotation of the alignment is done with the mouse. Keeping the left button pressed and moving the mouse rotates the alignment.
- The up/down and left/right arrow keys make the alignment move up/down or left/right.
- Zooming is done with the mouse wheel. Alternatively this can be done with the page up/page down keys if one does not have a mouse wheel.
- If one got lost in space while navigating through the alignment, just select *default view* in the *View settings* menu.

In the *exploration mode* the layout menu is empty and no new layout compuation can be done. Changing the mode again obviously allows new layout computations.

File menu

In this menu there are the *open* and the *quit* action and additionally two *settings* dialogs. All these actions can be used in both modes.

- *Open* opens a file dialog, the user has to choose a .sif file as specified above.
- *Quit* ends the program.
- *Settings* opens a dialog allowing to define some layout settings for the alignment. It consists of one tab for the general and one tab for network specific settings. In the tab for the general settings there are several options
  - *collapse paralogs* lets paralogous nodes be represented by just one node
  - *hide edges* causes drawing only the nodes without the edges
  - *show labels* causes the rendering of labels at each node
  - *background color* lets the user choose a background color
  - *node size* lets the user choose a size for the nodes
  - *text size* lets the user choose a text size for the labels
  - *distance between the different layers* lets the user change the distance between the layers if in 2.5D layout
  - *label dispersion* lets the user disperse the labels; moving the slider to the right causes the labels to move upward with respect to the node they belong to, middle position causes no displacement and left positions cause displacement downward. The displacement is always zero for the network in the background and increases for the networks towards the foreground. This allows the user to make the labels visible if they are cluttered.

- *Show inter-network edges* draws edges between all the orthologous proteins of the different networks, this can only be activated together with the side by side layout.

Additionally the layout type can be choosen, the user has the choice between *2.5D Layout*, *side by side*, and *all in one* layout.

The tab for the single networks looks nearly the same as the general tab, but the selection of options only changes the network in question. But there are a few additional options

- *hide complete network* makes this network invisible
- *node type* lets the user choose the type/geometry (ball, cube, diamond, pyramid) for the nodes of this network
- *node color* lets the user choose the color for the nodes

The button *Apply* activates all the changes made, *cancel* closes the dialog and does not change any settings. The *OK* button also closes the dialog but applies the new chosen settings.

- *SA Settings* action opens a dialog for changing the parameters for the simulated annealing algorithm (see below). Moving a slider to the right makes the respective property be punished stronger in the simulated annealing algorithm. Stronger punished properties will more likely be avoided.
  - *edge crossings* the number of edge crossings
  - *edge length* sum of edge lengths, to avoid very long edges
  - *edge angle* if this value is higher, there are fewer small angles between adjacent edges
  - *minimum node distance* prevents nodes to be too close together

Layout Menu

In layout computation mode the user is able to modify the layout of the alignment. Therefore, there are several layout algorithms, which can be selected in the *Layout* menu. Manually changing the layout by classical *drag and drop* using the left mouse button is also possible. Please collapse all paralogs (in the *settings* dialog) before moving them, otherwise the single nodes will be moved, which causes paralogs to be no longer nearby each other. The following layout algorithms are available:

- *Random* computes a random layout
- *Spring Embedder* computes a layout using Fruchterman and Reingold
- *Kamada Kawai* computes a layout using Kamada and Kawai

- *Simulated Annealing* computes a layout using Simulated Annealing with the parameters specified in the *SA Settings* dialog (see above). The simulated annealing algorithm is due to its complexity slower than the others. However, layout results are usually better. A progress dialog shows the progress of the algorithm and allows the user to abort it. The algorithm consists of several runs of the same number of loops and the number of runs is a priori unknown. Stopping the algorithm, makes it stop after the end of the current run.
- *Simulated Annealing Update* same as Simulated annealing but starting with the current layout and allowing only small changes. Thus this algorithm might be used when one already got a good layout, which should be improved.
- *undo* makes the manual changes be undone
- *open layout* opens a file dialog from which the user can choose a .pos file with a layout for this network. The network will have the layout stored there.
- *save layout* Once a pleesant layout is computed, one can store this layout in a .pos file, which can be opened in later sessions again. In this file the positions of the single nodes are stored.

Mode Menu

In this menu the user changes the mode.

- *Layout computation mode* changes into layout computation mode
- *Exploration mode* changes into exploration mode, this mode allows different changes in the viewpoint.

View Settings Menu

has got two menu points,

- *default view* which changes the viewpoint to the default one
- *search Protein* which opens a Dialog. When a valid Protein name is inserted in the dialog, this Protein is moved to the center, zooming in using the mouse wheel now zooms the found protein.

Screen Shot

This is shown with a camara icon and it opens a file dialog for storing the current view as a .png or .jpg image. Thus the user easily stores pictures of scenes he finds especially interesting.

The example data sets are original files computed by Sharan et al. [4], also the file names are given by them and we did not change them. These data sets are real world data sets.

References

[1] Shannon P, Markiel A, Ozier O, Baliga NS, Wang JT, Ramage D, Amin N, Schwikowski B, Ideker T: **Cytoscape: a software environment for integrated models of biomolecular interaction networks.** *Genome Res* 2003, **13**(11):2498–2504, [http://dx.doi.org/10.1101/gr.1239303].

[2] Kalaev M, Bafna V, Sharan R: **Fast and Accurate Alignment of Multiple Protein Networks.** In *RECOMB*, *Volume 4955 of* Lecture Notes in Computer Science. Edited by Vingron M, Wong L, Springer 2008:246–256, [http://dblp.uni-trier.de/db/conf/recomb/recomb2008.html#KalaevBS08].

[3] Kelley BP, Sharan R, Karp RM, Sittler T, Root DE, Stockwell BR, Ideker T: **Conserved pathways within bacteria and yeast as revealed by global protein network alignment.** *Proc Natl Acad Sci U S A* 2003, **100**(20):11394–11399, [http://dx.doi.org/10.1073/pnas.1534710100].

[4] Sharan R, Suthram S, Kelley R, Kuhn T, McCuine S, Uetz P, Sittler T, Karp R, Ideker T: **Conserved patterns of protein interaction in multiple species.** *Proc Natl Acad Sci U S A* 2005, **102**(6):1974–1979.
