# Supplementary material for: VANLO - Interactive visual exploration of aligned biological networks
Source: BMC Bioinformatics. 2009 Oct 12;10:327. doi: 10.1186/1471-2105-10-327 (PMC2766391; doi:10.1186/1471-2105-10-327)
Supplement: Additional file 2 — Manual. The file manual.pdf includes a manual for the use of the VANLO software and a file format specification for the input files used. [file 1471-2105-10-327-S2.PDF]

Background on Protein Protein Interaction Network Evolution

Steffen Brasch   Lars Linsen   Georg Fuellen

July 28, 2009

Proteins are linear polymers built from 20 building blocks called amino acids. Parts of a protein that are found conserved in many other proteins are called domains; these domains are usually responsible for the protein-protein interactions.

Protein-protein interactions (PPIs) are transient or permanent connections between proteins, and they are important for many biological phenomena such as signaling, transcriptional regulation, and multi-enzyme complexes. They are explained by molecular adhesive forces between parts of the proteins (domains) which in turn can be tracked down to the atomic level.

Interaction networks evolve by the loss and gain of nodes (proteins) and links (interactions). Biologists assume that the complex networks interconnecting the components of an organism such as a human being are, like all of life, the result of a more or less gradual evolutionary process of descent with modification. Emergence of biological complexity is nevertheless poorly understood, and a deeper understanding is of utmost importance.

The most simple kind of evolutionary scenario yielding a complex interaction network is depicted in Figure I. A single protein without any interaction partners except itself is the most simple network (Figure I a). This protein is duplicated (by duplication of the underlying genomic material, i.e. the gene), and we observe two identical proteins that interact with each other (Figure I b). Evolution goes on, and the proteins become gradually different in shape and function, and we observe a small network of two distinct proteins related by duplication; they are called *paralogs*, and inherited interactions are called paralogous. Further duplications give rise to a more complex network of paralogs (Figure I c); note that some interactions were lost after duplication. Assume that we have followed the evolution of our proteins and their network up to the common ancestor of, for example, human and fly. After *speciation*, entities evolve differently in the two lineages of descent that yield the two different species (Figure I d and d'). The proteins related by speciation are called *orthologs* and the inherited interactions and the networks themselves are called orthologous. Conservation of PPIs across species has been observed in many cases [1, 2, 3], for example for the transcriptional network which regulates the development of the heart. It is regulated by interactions which have been conserved at least since the last common ancestor of human and fly [4]. By comparing the networks of many species,

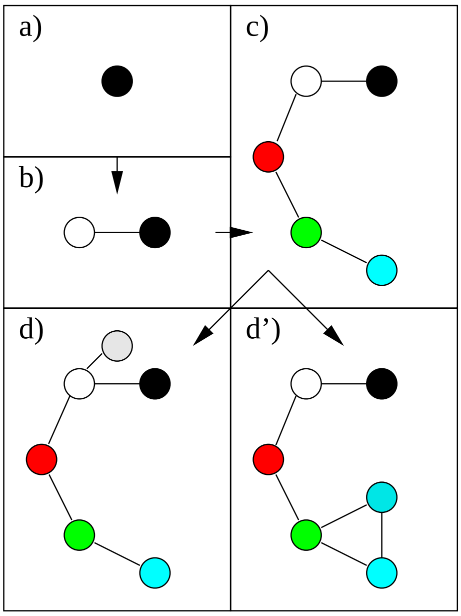

Figure I: Evolution of protein-protein interaction networks. A single protein a) duplicates and a small interaction network is built up b). For some time the network grows more complex as depicted in c). A speciation event splits the line of descent into two lineages d) and d'), that are evolving independently thereafter. In d)/d') all five proteins from c) are not understood as paralogous. However, in d), the white and the gray protein are paralogs, in d') the blue proteins are paralogs.

their ancestral core network can be estimated.

We do not know how precisely the PPI networks of life evolved. However, due to a lot of experimental work we know more and more PPI networks, or at least parts of them, for different species living today. Additionally, we can infer for many proteins in different species that they are orthologs, and for many proteins in the same species that they are paralogs.(Paralogs and orthologs are defined with respect to a reference point. In Figure I d) all proteins are paralogous with reference to the original protein in Figure I a). However, taking the proteins in Figure I c) as reference, the gray and the white protein as well as the blue proteins in Figure I d)/d') are paralogs. Reference points are defined by the set of proteins existing at a specific time.) Therefore we are able to align PPI networks. A *network alignment* for a number of networks from different species specifies which nodes (representing the proteins) in one network correspond to (i.e. are orthologous to) which nodes in the other networks. This correspondence may be one to one, or it may relate a set of paralogs in one species to an orthologous set of paralogs in another species. More precisely, we view proteins from one species to be paralogous if they evolved by duplication after the speciation event splitting the last common ancestor. Two proteins in one species that evolved from the same protein are *not* understood as paralogous *if* they were already distinct proteins in the PPI network of the last common ancestor. In other words, we

take the *last* speciation event as the reference point. For example in Figure I d) and d') the white and the black protein both evolved from the black protein in Figure I a) and thus could be viewed as paralogs. But we do not view them as paralogs, as they already where two distinct proteins in the common ancestor shown in Figure I c). For more than two networks the reference point is the last speciation event of the common ancestor of all aligned species. For the two networks in Figure I d) and d') the alignment is shown in Figure II.

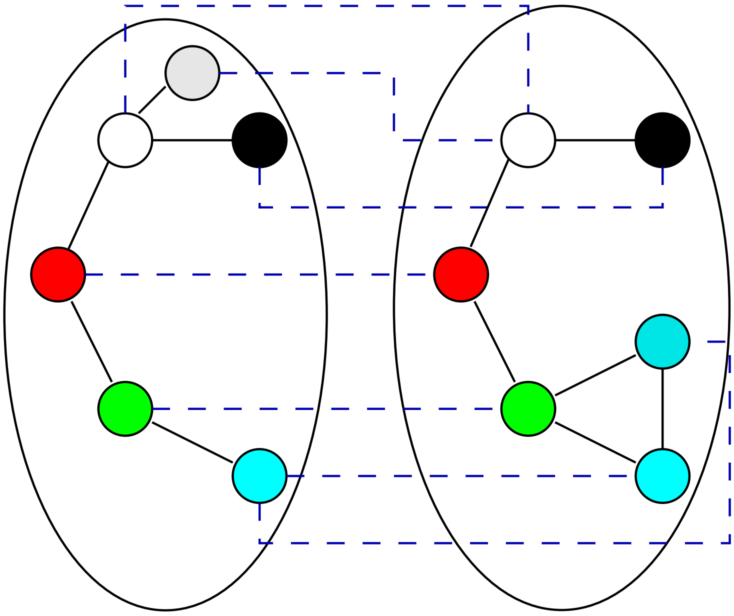

Figure II: Alignment of two PPI networks. In each ellipse the network of one species is shown, where the black, solid lines indicate the interactions between the proteins. The dashed blue lines show the alignment relation, i.e., two proteins connected by a dashed blue line are orthologous. Considering the transition from c) to d) and d') in Figure I, the white/gray and blue paralogs are orthologous to the same protein in the other species.

In biology, scientists are not only faced with PPI networks but with many other kinds of biological networks including regulatory ones that involve DNA-protein interaction and metabolic ones that include small metabolites as nodes. These networks are also related by evolution and can be aligned. Therefore visualization techniques developed for aligned PPI networks can also be used for these other kinds of biological networks. Analysis of all kinds of networks will gain importance, in particular in biomedicine. After all, complex diseases must be tackled nowadays: cancer, arteriosclerosis and dementia are all multifactorial. They all have their cause in the interplay of a multitude of factors, many of which corresponding to networks gone out of order. In this context, comprehensive visualization can be a trigger of medical progress.

References

[1] Bork P, Jensen L, von Mering C, Ramani A, Lee I, Marcotte E: **Protein interaction networks from yeast to human.** *Curr Opin Struct Biol* 2004, **14**(3):292–299.

[2] Ramani A, Marcotte E: **Exploiting the co-evolution of interacting proteins to discover interaction specificity.** *J Mol Biol* 2003, **327**:273–284.

[3] Matthews L, Vaglio P, Reboul J, Ge H, Davis B, Garrels J, Vincent S, Vidal M: **Identification of potential interaction networks using sequence-based searches for conserved protein-protein interactions or "interologs".** *Genome Res* 2001, **11**(12):2120–2126.

[4] Cripps R, Olson E: **Control of cardiac development by an evolutionarily conserved transcriptional network.** *Dev Biol* 2002, **246**:14–28.

[5] Sharan R, Ideker T: **Modeling cellular machinery through biological network comparison**. *Nature Biotechnology* 2006, **24**(4):427–433, [[http://dx.doi.org/10.1038/nbt1196]].

[6] Kelley BP, Sharan R, Karp RM, Sittler T, Root DE, Stockwell BR, Ideker T: **Conserved pathways within bacteria and yeast as revealed by global protein network alignment.** *Proc Natl Acad Sci U S A* 2003, **100**(20):11394–11399, [[http://dx.doi.org/10.1073/pnas.1534710100]].

[7] Sharan R, Suthram S, Kelley R, Kuhn T, McCuine S, Uetz P, Sittler T, Karp R, Ideker T: **Conserved patterns of protein interaction in multiple species.** *Proc Natl Acad Sci U S A* 2005, **102**(6):1974–1979.

[8] Berg J, Lässig M: **Local graph alignment and motif search in biological networks.** *Proc Natl Acad Sci U S A* 2004, **101**(41):14689–14694.

[9] Dutkowski J, Tiuryn J: **Identification of functional modules from conserved ancestral protein protein interactions**. *Bioinformatics* 2007, **23**(13):i149–158, [[http://bioinformatics.oxfordjournals.org/cgi/content/abstract/23/13/i149]].
